# Wideband Linearly Polarized Over-2-Bit Transmitarray Antenna for Millimeter-Wave Applications

**DOI:** 10.3390/mi17050605

**Published:** 2026-05-14

**Authors:** Yuanjun Shen, Xuli Feng, Tianling Zhang

**Affiliations:** 1 National Key Laboratory of Radar Detection and Sensing, Xidian University, Xi’an 710071, China; yuanjun.shen@xidian.edu.cn; 2 AVIC Shaanxi Aero Electric Co., Ltd., Xi’an 710065, China; xxllfeng@163.com

**Keywords:** over-2-bit, linearly polarized, transmitarray antenna, wideband

## Abstract

A wideband linearly polarized over-2-bit transmitarray antenna (TA) using the receiving-transmitting (R-T) scheme in the millimeter-wave band is presented in this work. The TA unit consists of two rectangular patches with a pair of bent branches, and the patches are connected by a metalized via. Two methods are used in this TA to obtain an over-2-bit phase shift of 0–90° and 180–270° from 18 GHz to 30 GHz. Firstly, 180° phase resolution is obtained by rotating the receiving patch around via by 180°. Secondly, by tuning the connection position between the branches and rectangular patch of the TA unit cell, a continuous 90° phase shift is further achieved. A TA prototype with 20×20 units is designed, fabricated, and measured. The measured 1 dB and 3 dB gain bandwidth is 24.9% (24.47–31.43 GHz) and 46.96% (20.45–33 GHz) respectively, with a peak gain of 25.17 dBi and a peak aperture efficiency of 55.2%. The measured results agree well with the simulated ones.

## 1. Introduction

As wireless communication technology advances, both radar and long-range communication systems require high-gain antennas to enhance the detection capability and communication distance of the entire system [1,2,3]. Transmitarray antennas (TAs) have garnered great interest due to their unique advantages, including high gain, low cost, simple feeding technique, no feed blockage, etc. [4,5,6]. A TA consists of a feeding source and a TA surface. The principle of TA is to convert a spherical incident wave radiated from the feeding source into the TA surface to form a planar outgoing wave and achieve a desired radiation pattern [7]. A focused beam in the specified direction can be achieved by controlling the transmission phase of TA units to compensate for the different path lengths from the feed source. Ideally, a continuous phase shift of 0–360° is desirable for a TA unit but achieving a seamlessly continuous 360° tuning ability over a broad frequency range with a straightforward structure appears to be challenging.

Recently, quantization phase compensation has been implemented in TAs because of its low complexity and low loss characteristic. Different configurations, such as 1-bit, 2-bit, and 3-bit TAs, have been extensively explored, as outlined in [8,9,10,11,12,13,14,15,16]. These configurations can be broadly categorized into two types. The first type uses multilayer frequency selective surfaces (M-FSSs), as discussed in [11,12,13]. In [13], two types of units with different configurations based on FSS were proposed with five metal layers and four substrate layers. The 2-bit phase shift is achieved by rotating the fourth metal layer based on polarization-rotating properties.

Another TA type employs the “receive–transmit (R-T)” scheme, where phase shifts are accomplished through the rotation of either the receiving or transmitting units [14,15]. Alternatively, phase adjustments can be achieved by changing the length of the phase delay lines [16,17,18], or using different units [19,20]. In [14], a wideband 1-bit TA based on a magneto-electric dipole unit was proposed, but 3.8 dB gain loss is inevitable due to large phase errors [11,21]. Subsequently, in [16], a 2-bit magneto-electric dipole TA was presented, which achieves a 3 dB gain bandwidth of 38.7% and a maximum aperture efficiency of 53%. The only drawback is that the phase delay line between the receiving and the transmitting units makes the structure complex. In [19], two different units were proposed to offer a 3-bit phase by changing the dimensions of the two units. The 1 dB and 3 dB gain bandwidths are 15.4% and 20%. However, the use of two types of units to implement a 3-bit quantization scheme results in a complicated design.

In this letter, we propose a wideband over-2-bit TA. The unit consists of two rectangular patches with a pair of bent branches. Two patches are printed on the substrate back-to-back and connected by a metalized via with a separated ground plane. By rotating the receiving patch around via by 180°, the phase shift of 180° is obtained. In addition, by tuning the connection position between the branches and rectangular patch of the receiving patch and the transmitting patch, the other continuous 90° phase shift can be achieved. The proposed topology unit can provide an over-2-bit continuous phase shift covering 0–90° and 180–270° from 18 GHz to 30 GHz with low insertion loss and wide bandwidth. The unit periodicity is 0.32λ, λ is the free space wavelength at 24 GHz, and the total thickness of the unit is 0.18λ.

## 2. Design and Simulation of TA

**Figure 1:**
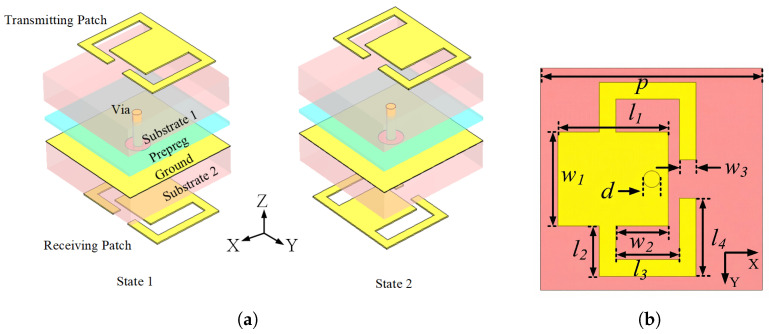
Configuration of the proposed TA unit. (**a**) 3D view. (**b**) Top view. The yellow regions denote metallic layers.

The structure of the proposed TA unit is shown in Figure 1. The TA unit consists of three metal layers, two identical substrates (Taconic TSM-DS3 (Taconic, Petersburgh, NY, USA), $\varepsilon_{r}=3$, tanδ=0.0014) and the total thickness of the unit is 0.18λ, where λ is the wavelength in free space at 24 GHz. The substrates are bonded by a glue layer (FR27-0040-43F (AGC Multi Material America, Inc., Tempe, AZ, USA), $\varepsilon_{r}=2.79$, tanδ=0.0014) with a thickness of 0.1 mm. The ground layer has a thickness of 0.018 mm. The receiving patch and transmitting patch are printed on the top and the bottom of the unit, respectively. Both patches have the same topology. They are connected by a metalized via to realize the transmission of electromagnetic energy. The period of the unit cell is 4 mm (0.32λ at 24 GHz, where λ is the wavelength in free space at this frequency). Dimensions of the units are given as follows: p=4 mm, $h_{s}=1.016$ mm, $l_{1}=1.7$ mm, $w_{1}=1.7$ mm, $l_{2}=0.9$ mm, $w_{2}=0.65$ mm, $l_{3}=1.15$ mm, $l_{4}=1.4$ mm, $w_{3}=0.3$ mm, and d=0.3 mm.

**Figure 2:**
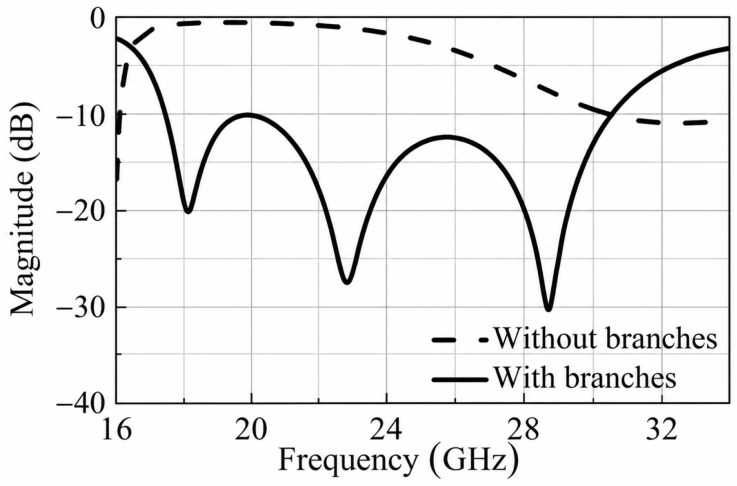
Reflection coefficients $|S_{11}|$ for patch with and without branches.

**Figure 3:**
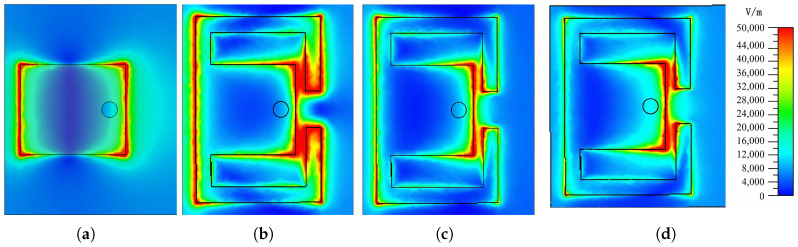
E-field distribution of the proposed patch (**a**) without branches at 24 GHz and with branches at frequency (**b**) 18.1 GHz, (**c**) 22.81 GHz, and (**d**) 28.745 GHz.

**Figure 4:**
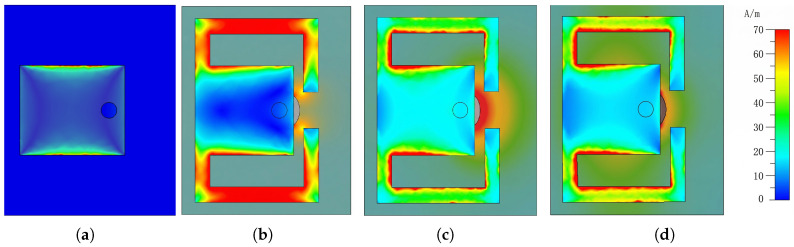
Surface current distribution of the proposed patch (**a**) without branches at 24 GHz and with branches at frequency (**b**) 18.1 GHz, (**c**) 22.81 GHz, and (**d**) 28.745 GHz.

Typically, microstrip patch antennas have a narrow bandwidth. To overcome this limitation and widen the bandwidth, a couple of bent branches are introduced. The reflection coefficient shown in Figure 2 indicates that the introduced bent branches generate additional resonant points and improve the overall reflection response. The electric field distribution of the receiving patch is depicted in Figure 3 which corresponds to the three resonance points described by the S-parameter. It is seen that the electric field is concentrated at the edge of the whole patch on the left and the gap between the branches and patch. Such an electric field distribution is attributed to the coupling between the rectangular patch and its two sides of the curved branches. Further analyzing the current distribution which is shown in Figure 4, the current is concentrated on the branch and the connection between the rectangular patch and the branch. It can be inferred that adjusting the position of the joint between patch and branches results in a change in the current path and the realization of a phase delay, which, in turn, achieves a continuous phase shift.

**Figure 5:**
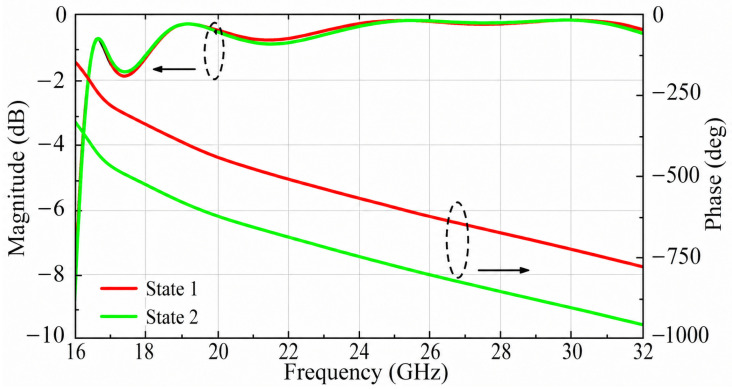
Transmission coefficients $|S_{21}|$ and phase of State 1 and State 2 ($w_{2}=1$ mm).

There are two states of the proposed unit, as shown in Figure 1a. In State 1, the receiving patch branches bend toward the x-axis, whereas, in State 2, the receiving patch branches bend toward the negative x-axis. By rotating the receiving patch by 180° around the z-axis, a phase difference close to 180° can be obtained between the two states. As shown in Figure 5, the two states exhibit similar transmission magnitudes and a stable phase difference close to 180° over the operating band. Specifically, both states exhibit a transmission loss of less than 1 dB from 18.2 to 32 GHz, and the maximum magnitude deviation between the two states is less than 0.2 dB within this frequency range. Therefore, the two states can serve as a practical 1-bit phase pair for the proposed over-2-bit phase assignment strategy.

**Figure 6:**
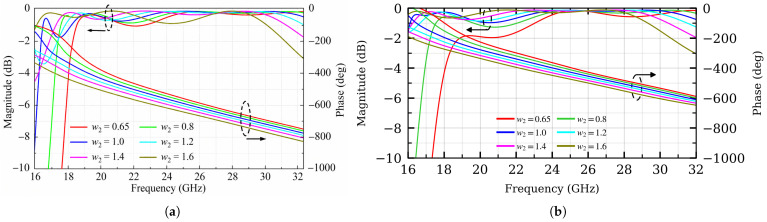
Transmission magnitude $|S_{21}|$ and phase with different values of $w_{2}$. (**a**) State 1. (**b**) State 2.

**Figure 7:**
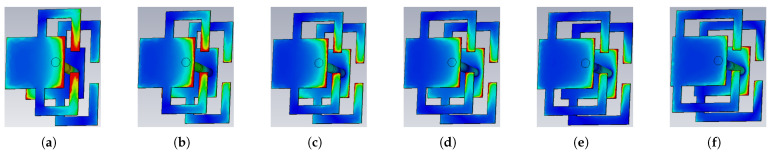
E-field distribution at 24 GHz of the proposed element under State 1 configuration with different values of $w_{2}$: (**a**) 0.65 mm; (**b**) 0.8 mm; (**c**) 1.0 mm; (**d**) 1.2 mm; (**e**) 1.4 mm; (**f**) 1.6 mm. The color map is the same with one used in Figure 3.

**Figure 8:**
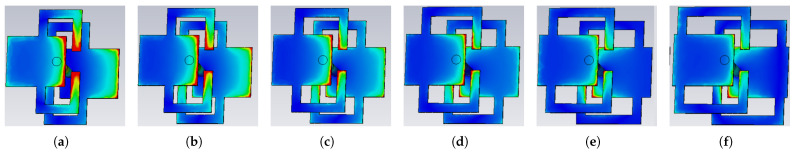
E-field distribution at 24 GHz of the proposed element under State 2 configuration with different values of $w_{2}$: (**a**) 0.65 mm; (**b**) 0.8 mm; (**c**) 1.0 mm; (**d**) 1.2 mm; (**e**) 1.4 mm; (**f**) 1.6 mm. The color map is the same with one used in Figure 3.

Figure 6 shows the simulated transmission magnitude and phase responses with different values of $w_{2}$ for both State 1 and State 2. In State 1, the unit achieves an approximately continuous 90° phase shift as $w_{2}$ varies from 0.65 mm to 1.6 mm, while maintaining a transmission magnitude higher than −1.1 dB from 18.2 to 30 GHz. In State 2, a similar phase-tuning behavior is observed, with the transmission magnitude better than −2 dB from 18.8 to 30.5 GHz. These results confirm that both states can provide the required phase-tuning capability with acceptable transmission loss for the proposed over-2-bit TA design. Figure 7 and Figure 8 show the E-field distributions of the unit cell under both states with different values of $w_{2}$. As $w_{2}$ varies, the field concentration around the bent branches and their connection region changes accordingly, indicating that the local coupling path is modified. This explains the transmission-phase tuning behavior observed in Figure 6.

**Figure 9:**
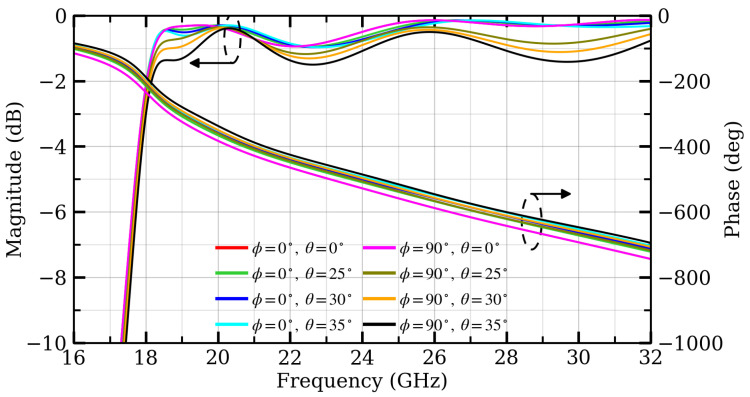
Transmission magnitude $|S_{21}|$ and phase for different oblique incident angles with $w_{2}=1$ mm in State 1.

Figure 9 shows the simulated transmission magnitude and phase responses of the proposed unit under different oblique incidence angles. Since the TA unit is illuminated by the feed horn at a finite focal distance, different unit cells across the aperture experience different local incidence angles. Therefore, the angular stability of the unit-cell transmission response should be evaluated.

In the proposed TA, the aperture contains 20×20 unit cells with a period of 4 mm, and the focal distance is 80 mm. The local incidence angle can be estimated as

(1) $$\theta \left(x,y\right)=\tan^{-1}\left(\frac{\sqrt{x^{2}+y^{2}}}{F}\right),$$

where *F* is the focal distance. For the outermost unit-cell centers in the principal planes, the maximum lateral distance from the aperture center is approximately 9.5p=38 mm. Therefore, the maximum incidence angle in the E- and H-plane edge regions is $\theta_{\mathrm{edge}}=\tan^{-1}\left(38/80\right)=25.4{}^{\circ}$. For this reason, the results within 25° are first focused on, since they represent the dominant principal-plane edge illumination condition of the proposed F/D = 1 configuration.

As shown in Figure 9, when the incidence angle is within 25°, the transmission magnitude remains higher than approximately −1.1 dB, and the phase deviation is within approximately 25°. This indicates that the proposed unit maintains acceptable transmission magnitude and phase stability over the main incidence-angle range associated with the central and principal-plane edge regions of the aperture.

Larger incidence angles can occur near the aperture corners. The corner incidence angle estimated from the outermost unit-cell centers is $\theta_{\mathrm{corner}}=\tan^{-1}\left(\sqrt{2}\times 38/80\right)=33.9{}^{\circ}$, and it is close to 35° if the physical aperture boundary is considered. Therefore, additional oblique-incidence results at 30° and 35° are also included in Figure 9 to further clarify the angular limitation of the unit cell. The transmission magnitude remains higher than approximately −1.4 dB and −1.7 dB for incidence angles up to 30° and 35°, respectively. Meanwhile, the phase deviation increases to about 35° at 30° and further increases to about 50° at 35°.

These results show that the unit-cell response gradually deteriorates under stronger oblique incidence, especially in terms of phase stability. However, the larger incidence angles mainly correspond to the near-corner region of the aperture rather than the dominant principal-plane edge region. These corner elements are generally more weakly illuminated by the feed horn due to the feed taper and the longer propagation distance. Consequently, their contribution to the main-beam formation is less significant than that of the central and principal-plane edge elements. Therefore, although the response degradation at 35° is acknowledged, the 0–25° range remains the primary incidence-angle range for evaluating the proposed TA unit, while the additional 30° and 35° results make the oblique-incidence analysis more complete.

## 3. TA Design and Results

The required continuous phase distribution of the TA aperture is calculated at 24 GHz according to the phase compensation method in [8]. For broadside radiation, the required compensation phase of the (m,n)-th element can be expressed as

(2) $$\phi_{\mathrm{req}}\left(m,n\right)={\mathrm{wrap}}_{0{}^{\circ}-360{}^{\circ}}\left[k_{0}\left(R_{m,n}-F\right)\frac{180{}^{\circ}}{\pi }\right],$$

where $k_{0}$ is the free-space wavenumber at 24 GHz, *F* is the focal distance, and $R_{m,n}$ is the distance between the feed phase center and the (m,n)-th unit cell.

To implement the required continuous phase distribution using the proposed unit, an over-2-bit phase mapping strategy is adopted. It should be noted that this strategy is not a conventional uniform 2-bit quantization scheme. Instead, it maps the required continuous phase to the physically available phase-tuning ranges of the proposed R–T unit. The phase assignment rule is expressed as

(3) $$\left\{\begin{array}{c} 0{}^{\circ}\leq \phi_{\mathrm{req}}<90{}^{\circ}\to \phi_{\mathrm{ass}}=\phi_{\mathrm{req}}\\ 90{}^{\circ}\leq \phi_{\mathrm{req}}<180{}^{\circ}\to \phi_{\mathrm{ass}}=90{}^{\circ}\\ 180{}^{\circ}\leq \phi_{\mathrm{req}}<270{}^{\circ}\to \phi_{\mathrm{ass}}=\phi_{\mathrm{req}}\\ 270{}^{\circ}\leq \phi_{\mathrm{req}}<360{}^{\circ}\to \phi_{\mathrm{ass}}=270{}^{\circ} \end{array}\right.$$

where $\phi_{\mathrm{req}}$ is the required continuous phase and $\phi_{\mathrm{ass}}$ is the assigned phase after the over-2-bit mapping.

**Figure 10:**
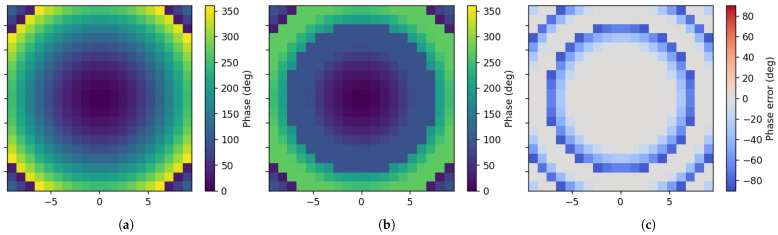
Phase distribution of the proposed TA at 24 GHz. (**a**) Required continuous phase distribution. (**b**) Implemented over-2-bit phase distribution. (**c**) Phase error distribution of the over-2-bit phase assignment.

The calculated required continuous phase distribution is shown in Figure 10a. Based on the proposed phase mapping strategy in Equation (3), the implementable over-2-bit phase distribution is obtained, as shown in Figure 10b. The corresponding phase error distribution is shown in Figure 10c. The phase error is defined as the wrapped phase difference between the assigned phase and the required continuous phase:

(4) $$\Delta \phi_{m,n}={\mathrm{wrap}}_{-180{}^{\circ}-180{}^{\circ}}\left[\phi_{\mathrm{ass}}\left(m,n\right)-\phi_{\mathrm{req}}\left(m,n\right)\right].$$

As shown in Figure 10c, the phase error is mainly introduced in the phase regions that cannot be continuously covered by the proposed unit, namely 90–180° and 270–360°. In the available phase-tuning regions of 0–90° and 180–270°, the required phase can be directly implemented with much smaller mapping error.

To further evaluate the influence of the phase mapping on the aperture performance, the phase efficiency can be estimated as

(5) $$\eta_{\phi }=\frac{{\left|\sum_{m,n}A_{m,n}e^{j\Delta \phi_{m,n}}\right|}^{2}}{{\left|\sum_{m,n}A_{m,n}\right|}^{2}},$$

where $A_{m,n}$ is the aperture amplitude weight of the (m,n)-th element, including the feed illumination and the transmission magnitude of the unit cell. In Equation (5), $\Delta \phi_{m,n}$ is converted to radians. The corresponding phase-related gain loss is calculated as

(6) $$L_{\phi }=-10\log_{10}\left(\eta_{\phi }\right).$$

For a standard uniform 2-bit quantization scheme, the phase error is usually assumed to be uniformly distributed within ±45°. The corresponding theoretical phase efficiency is

(7) $$\eta_{\phi ,2\mathrm{bit}}={\left[\frac{\sin(\pi /4)}{\pi /4}\right]}^{2}=0.8106,$$

which corresponds to a phase-related gain loss of 0.91 dB.

For the proposed over-2-bit phase mapping, the 0–90° and 180–270° regions provide continuous phase tuning, whereas the 90–180° and 270–360° regions are mapped to fixed phase states. Under a conservative assumption that the required phase is uniformly distributed over 0–360°, the phase efficiency can be estimated as

(8) $$\eta_{\phi ,\mathrm{prop}}={\left|\frac{\pi +2\int_{0}^{\pi /2}e^{-ju}\;du}{2\pi }\right|}^{2}=0.7710,$$

corresponding to a phase-related gain loss of 1.13 dB. Therefore, under the uniform phase-distribution assumption, the proposed non-uniform mapping introduces about 0.22 dB larger phase-related gain loss than standard uniform 2-bit quantization. This confirms that the proposed phase mapping should be evaluated based on the actual aperture phase and amplitude distribution, rather than only by its nominal phase resolution.

In a practical transmitarray, the feed illumination is tapered and the aperture elements do not contribute equally to the main beam. Therefore, the effective phase-related gain loss depends on the weighted aperture distribution. If the weighted proportion of the elements falling into the fixed phase regions is denoted as *p*, it can be expressed as

(9) $$p=\frac{\sum_{\mathrm{fixed}\;\mathrm{regions}}A_{m,n}}{\sum_{\mathrm{all}\;\mathrm{elements}}A_{m,n}}.$$

The phase efficiency of the proposed mapping can then be approximated as

(10) $$\eta_{\phi }\left(p\right)={\left|\left(1-p\right)+p\frac{2}{\pi }\left(1-j\right)\right|}^{2}.$$

Several calculated reference values are summarized in Table 1. These results indicate that the phase-related loss of the proposed over-2-bit mapping is aperture-distribution-dependent. The proposed strategy is therefore not claimed to be universally superior to standard uniform 2-bit quantization for arbitrary phase distributions. Instead, its practical advantage lies in providing additional continuous phase-control degrees of freedom in the available phase regions while maintaining a simple and low-loss R–T unit-cell structure.

**Figure 11:**
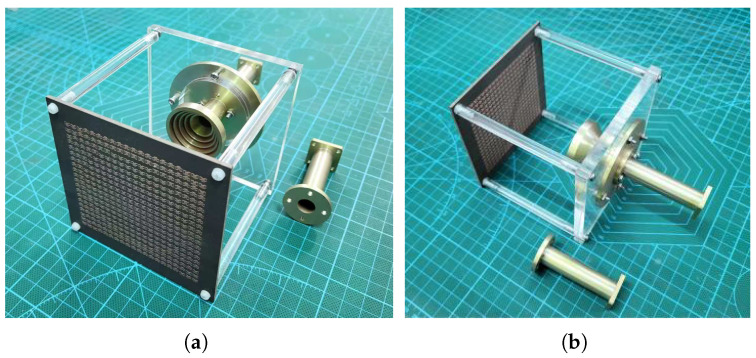
Photographs of the fabricated TA prototype. (**a**) Front view. (**b**) Back view.

After calculating the required phase distribution and applying the proposed over-2-bit phase assignment strategy, a TA prototype with 20×20 unit cells was designed and fabricated. The front and back views of the fabricated prototype are shown in Figure 11a and Figure 11b, respectively. The TA aperture is supported by an acrylic fixture, and the feed horn is placed 80 mm away from the center of the array, corresponding to an F/D ratio of 1.

**Figure 12:**
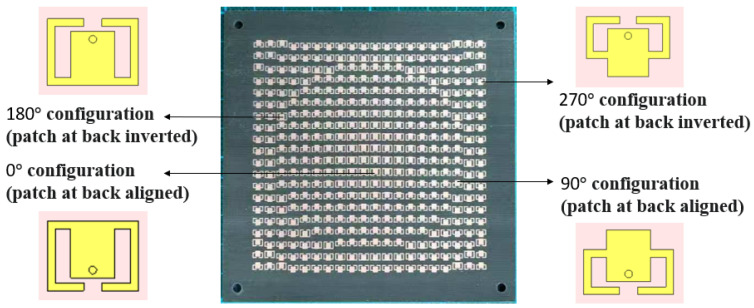
Transmitting-side view of the fabricated TA, with representative unit-cell configurations highlighted for the four reference phase states of 0°, 90°, 180°, and 270°.

To further clarify the physical implementation of the phase distribution, the transmitting side of the fabricated TA is shown in Figure 12, where representative unit-cell configurations corresponding to the four reference phase states of 0°, 90°, 180°, and 270° are highlighted. The 0° and 90° configurations are implemented with the patch on the opposite side aligned, while the 180° and 270° configurations are obtained by inverting the patch on the opposite side. Therefore, Figure 12 provides a direct visual correspondence between the phase assignment strategy and the fabricated aperture layout.

**Figure 13:**
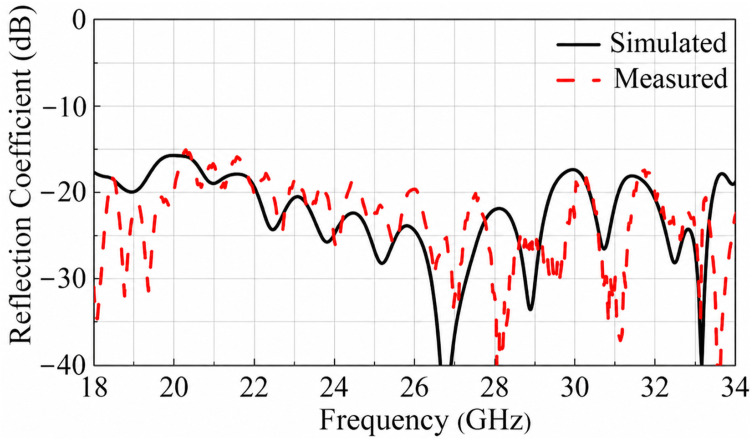
Simulated and measured reflection coefficients $|S_{11}|$ of the proposed TA.

**Figure 14:**
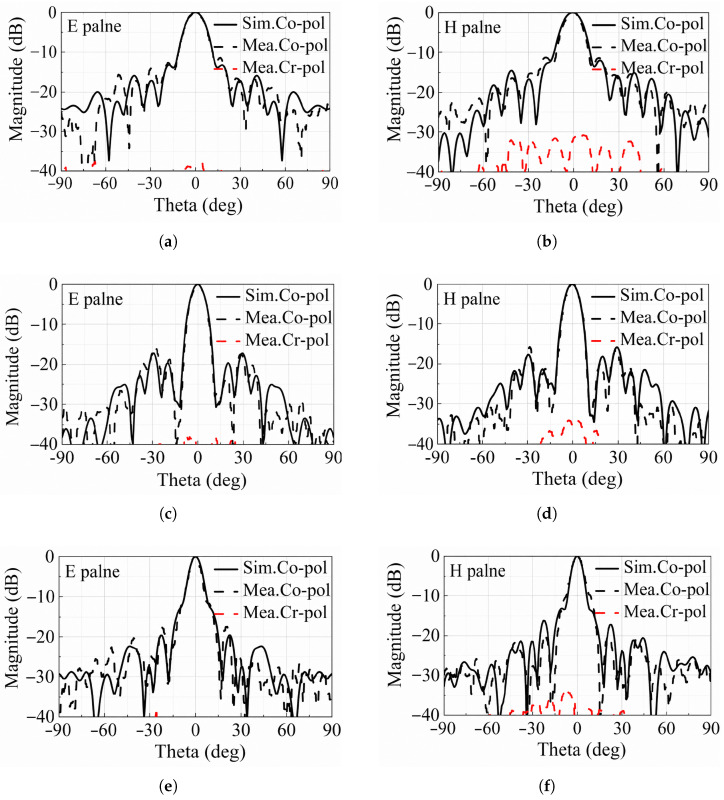
Comparison of the measured and simulated normalized radiation patterns at different frequencies. (**a**) E-plane and (**b**) H-plane at 19 GHz; (**c**) E-plane and (**d**) H-plane at 24 GHz; (**e**) E-plane and (**f**) H-plane at 29 GHz.

The simulated and measured reflection coefficients are represented in Figure 13 and a good agreement can be observed. Figure 14 shows the simulated and measured normalized radiation patterns of the TA in both the E- and H-plane at 19, 24, and 29 GHz. An excellent agreement is observed between simulations and measurements. At 24 GHz, the measured sidelobe levels (SLLs) are less than −15.76 dB in the E-plane and −15.8 dB in the H-plane. In addition, the measured cross-polarization level of the TA is below −30 dB in both the E-plane and H-plane.

**Figure 15:**
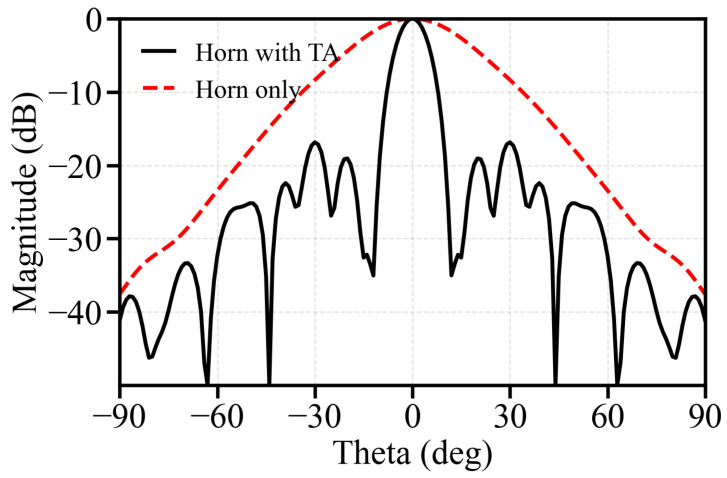
Simulated radiation pattern comparison of the feed horn alone and the feed horn loaded with the proposed TA at 24 GHz.

To further illustrate the function of the proposed transmitarray, Figure 15 compares the simulated radiation patterns of the feed horn alone and the feed horn loaded with the proposed TA at 24 GHz. It can be observed that the standalone horn exhibits a relatively broad beam, with a peak gain of 14.5 dBi and a 3 dB beamwidth of 32°. After loading the proposed TA, the radiation is effectively collimated into a much narrower main beam, and the peak gain is increased to 24.2 dBi, while the 3 dB beamwidth is reduced to 9.1°. These results clearly demonstrate that the proposed TA transforms the spherical-like incident wave radiated by the horn into a highly directive beam, thereby significantly improving the radiation directivity and gain performance.

**Figure 16:**
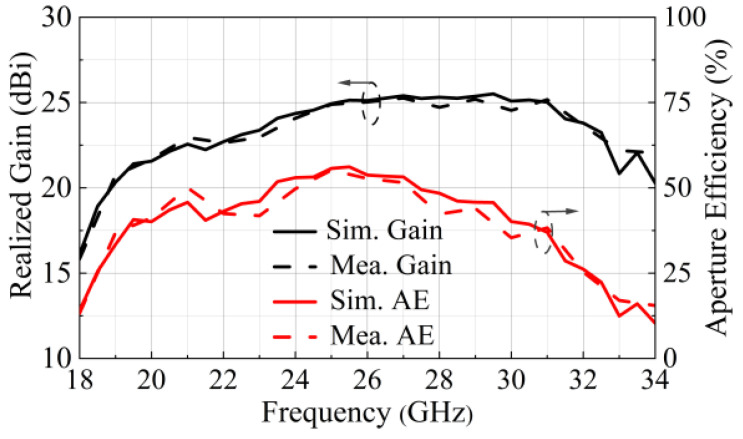
Simulated and measured gains and AE of the proposed TA.

The gain enhancement introduced by the proposed TA is further quantified in Figure 16, which shows the simulated and measured gain and aperture efficiency of the TA. The aperture efficiency (AE) is calculated using the formula given in [20]. Measured results show that the peak realized gain is 25.17 dBi at 31 GHz while peak aperture efficiency is 55.2% and occurred at 25 GHz. Gain bandwidths at 1 dB and 3 dB are 24.9% (24.47–31.43 GHz) and 46.96% (20.45–33 GHz).

Table 2 illustrates the main performance of the proposed TA with other TAs of the previous works. The proposed over-2-bit TA can achieve a high aperture efficiency which is higher than TA in [16], slightly. Compared with the 3-bit [19,20] or continuous phase compensation schemes [4,22,23], the proposed TA aperture efficiency is competitive. Additionally, the proposed TA also obtains a wider 3 dB gain bandwidth. To summarize, the proposed wideband over-2-bit TA obtains the highest aperture efficiency and widest 3 dB gain bandwidth simultaneously with low complexity. Due to the good features of low cost, low profile and wide bandwidth, the proposed TA can be a good candidate for potential applications in modern millimeter-wave wireless communication systems.

## 4. Conclusions

A wideband linearly polarized over-2-bit TA using an R-T structure is proposed in this work. The TA unit consists of three metal layers and two identical substrates. Two methods are used to achieve over-2-bit phase shifting units covering 0–90° and 180–270° from 18 GHz to 30 GHz. A 20×20 unit TA prototype has been fabricated and measured and the measured results closely match the simulated results. The measured results indicate that the 1 dB and 3 dB gain bandwidths are 24.9% and 46.96%, with a peak gain of 25.17 dBi. Furthermore, the measured peak aperture efficiency reaches 55.2%. The proposed TA is a good candidate for millimeter-wave applications due to its wide bandwidth and stable radiation performance.

## Figures and Tables

**Table 1:** Estimated phase-related gain loss for different phase assignment cases.

Phase Assignment Case	Phase Efficiency	Phase-Related Gain Loss
Ideal continuous phase	1.0000	0 dB
Standard uniform 2-bit	0.8106	0.91 dB
Proposed over-2-bit, uniform phase	0.7710	1.13 dB
Proposed over-2-bit, p=0.35	0.8115	0.91 dB
Proposed over-2-bit, p=0.30	0.8303	0.81 dB
Proposed over-2-bit, p=0.25	0.8519	0.70 dB

**Table 2:** Comparison of the proposed TA with reported transmitarray antennas.

Ref.	Center Freq. (GHz)	Element Type	F/D	Thickness ($\lambda_{0}$)	Aperture Size ($\lambda_{0}$)	Peak Gain (dBi)	1 dB/3 dB Gain BW (%)	Peak AE (%)	Phase Range	No. of Metal Layers
[4]	11.3	M-FSS	0.8	0.51	$\pi \times 8.1^{2}$	28.9	9/19.4	30 *	360	3
[13]	30	M-FSS	0.823	0.2	8.5 × 8.5	26.1	29/43.7	44.7	2BIT	5
[14]	27	R-T	1.07	0.198	8.6 × 8.6	24.5	-/-	28	1BIT	6
[15]	27	R-T	1.07	0.198	8.3 × 8.3	26.8	-/33	41	OVER-2-BIT	8
[16]	23	R-T	1	0.21	6.4 × 6.4	25.7	-/38.7	53	2BIT	5
[18]	10	R-T	0.8	0.11	7.5 × 7.5	24.7	-/22.8	49	2BIT	4
[19]	61.5	R-T	0.7	0.23	$\pi \times 10.25^{2}$	32.5	15.4/-	42.7	3BIT	3
[20]	145	R-T	0.75	0.24	20 × 20	33	11.7/-	38.3	3BIT	3
[22]	21	R-T	1.5	0.14	13.23 × 13.23	29.4	9.6/16.7	40 *	360	2
[23]	14	R-T	0.83	0.13	5.6 × 5.6	22.2	18.7/-	50.7	360	4
Pro.	24	R-T	1	0.18	6.4 × 6.4	25.17	24.9/46.96	55.2	over-2-bit	3

* The aperture efficiency is calculated based on the maximum gain and the aperture dimensions available.

## Data Availability

Data are contained within the article.

## Abbreviations

The following abbreviations are used in this manuscript:

TA	Transmitarray antenna
R-T	Receive–transmit
M-FSS	Multilayer frequency selective surface
FSS	Frequency selective surface
AE	Aperture efficiency
SLL	Sidelobe level
